# Incorporating Virtual Reactions into a Logic-based Ligand-based Virtual Screening Method to Discover New Leads

**DOI:** 10.1002/minf.201400162

**Published:** 2015-03-20

**Authors:** Christopher R Reynolds, Stephen H Muggleton, Michael J E Sternberg

**Affiliations:** [a] Department of Bioinformatics, Imperial College London, South Kensington Campus London SW7 2AZ, UK; [b] Department of Computing, Imperial College London, South Kensington Campus London SW7 2AZ, UK

**Keywords:** Virtual screening, Reactions, Ligand, Synthesis, Machine learning

## Abstract

The use of virtual screening has become increasingly central to the drug development pipeline, with ligand-based virtual screening used to screen databases of compounds to predict their bioactivity against a target. These databases can only represent a small fraction of chemical space, and this paper describes a method of exploring synthetic space by applying virtual reactions to promising compounds within a database, and generating focussed libraries of predicted derivatives. A ligand-based virtual screening tool Investigational Novel Drug Discovery by Example (INDDEx) is used as the basis for a system of virtual reactions. The use of virtual reactions is estimated to open up a potential space of 1.21×10^12^ potential molecules. A de novo design algorithm known as Partial Logical-Rule Reactant Selection (PLoRRS) is introduced and incorporated into the INDDEx methodology. PLoRRS uses logical rules from the INDDEx model to select reactants for the de novo generation of potentially active products. The PLoRRS method is found to increase significantly the likelihood of retrieving molecules similar to known actives with a *p*-value of 0.016. Case studies demonstrate that the virtual reactions produce molecules highly similar to known actives, including known blockbuster drugs.

## 1 Introduction

Virtual screening methods search databases of molecular structures for “hits” which synthetic chemists can then explore by modifying the hits to develop them into lead series. Small-molecule space has been estimated to contain 10^63^ molecules,[1] and the subset of drug-like small-molecules at between 10^14^ and 10^30^ molecules.[2] This precludes the tractability of a brute-force search through these spaces. Even the largest databases, such as the GDB-17 database of 1.66×10^11^ molecules containing up to 17 atoms[3] can only cover a fraction of this space. Previous virtual screening efforts involving INDDEx[4] have focused on searching the ZINC database of over 35 million purchasable compounds.[5,6] Another disadvantage of just searching databases is that there can also be leaps between hit and lead activity so potentially active lead series can be missed by prioritizing only the hits with the highest predicted activities. The cost and effort of synthesis are further concerns when developing leads. There is therefore a requirement for an in silico method to predict the activity of leads rather than hits.

One method to explore more of chemical space is to generate unfocussed combinatorial virtual libraries of billions of synthesisable molecules to screen, such as InhibOx’s VSPACE[7] which uses ChemAxon’s virtual reaction toolkits.[8] An alternative approach is to use de novo design algorithms, which assemble novel molecules from atoms or fragments rather than scanning libraries of molecules. Methods that incorporate de novo design into virtual screening include PRO_SELECT,[9] DREAM++[10] and TOPAS.[11] TRIPOS provides tools for de novo design as part of its SYBYL-X suite,[12] and Schrodinger provides tools as part of its Glide software.[13]

When exploring synthetic space, a balance must be found that provides sufficient recall to generate enough products to have a reasonable chance of detecting a true positive active whilst having a predictive method that is precise enough to keep the number of false positives below a level where it would still be feasible to synthesise and test all positives.

INDDEx is a drug discovery technology that performs ligand-based virtual screening for drug discovery and was previously described in Reynolds et al.[14] It uses the supervised machine-learning technique Support Vector Inductive Logic Programming (SVILP),[15, 16] which integrates the Inductive Logic Programming (ILP) technique,[17] and Support Vector Machine (SVM) technique.[18] INDDEx learns logical rules determining activity and inactivity from a dataset of active and inactive molecules, and an SVM is used to weight the rules. Reynolds et al.[14] benchmarked the performance of INDDEx at retrieving molecules on the Directory of Useful Decoys (DUD)[19] and found INDDEx outperformed other the screening methods eHiTS LASSO,[20] PharmaGist[21] and DOCK:[22] training on eight ligands, INDDEx achieved mean Enrichment Factors of 90.4 and 707 on 1 % and 0.1 % of the database respectively, and an Enrichment Factor of 66.9 on 1 % of the database when excluding all similar ligands (defined as ligands with an MCSS (Maximum Common Substructure) Tanimoto coefficient≥0.5 to any of the ligands in the training data). In addition, SVILP has been used for molecular toxicology prediction[15] and the search for SIRT2 inhibitors.[4]

## 2 Methods

The approach develops INDDEx by using the rules generated by the SVILP to select potential reactants to participate in virtual reactions to generate potential leads. The rules for the virtual reactions are taken from ChemAxon’s reaction toolkits (JChem version 5.2.0, 2009),[8] and a list of the virtual reactions used is given in Appendix A of the supporting information.

### 2.1 INDDEx Method

**Figure 1:**
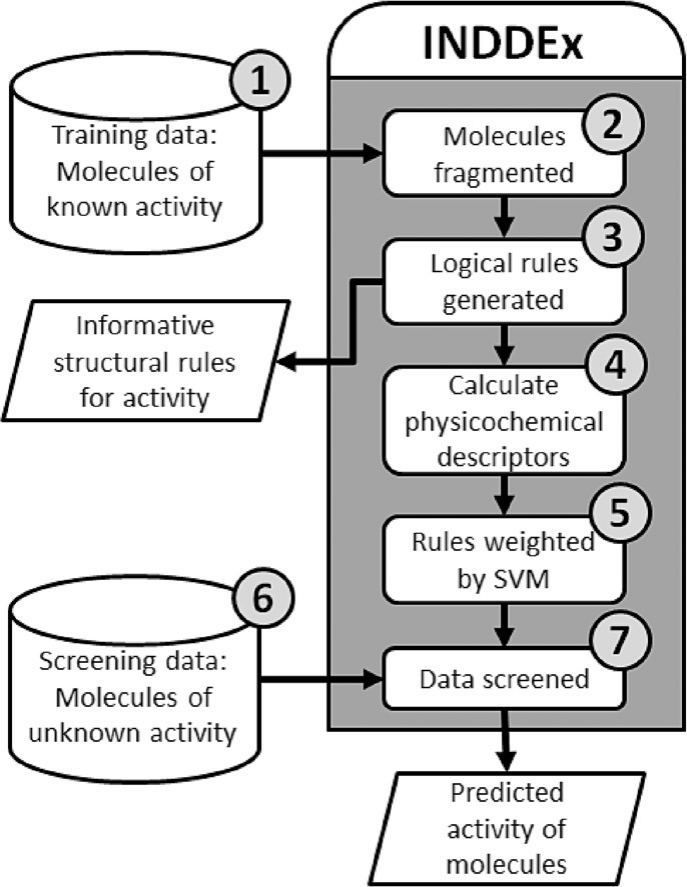
Diagram of INDDEx method incorporating SVILP.

Figure 1 shows activity prediction process performed by the INDDEx method incorporating SVILP. The cylinders represent the datasets used and the grey-shaded area indicates the processes performed by the program. These are discussed in detail below. This is the method previously described in Reynolds et al.[14]

1. INDDEx performs supervised machine-learning from a set of training data. This is comprised of active and inactive molecules in an energetically minimized 3D structure, with active molecules assigned a measure of activity. The minimization field used throughout this paper is the MMFF94 Merck molecular force field.[23]
2. Each molecule is decomposed into a group of fragments; a series of 2D strings representing molecular substructure. Each heavy atom in a molecule forms the central atom of a fragment. A fragment consists of the central heavy atom element (ignoring hybridisation), all immediately neighbouring heavy atom elements and the bond types connecting them and the total number of hydrogen atoms connected to the central atom and immediate neighbours. For example, the oxygen in an acetaldehyde forms the fragment “Oxygen, connected to carbon by a double bond, hydrogenation of one,” and each carbon in a benzene ring forms the fragment “Carbon atom, connected to carbon by an aromatic bond, connected to another carbon by an aromatic bond, hydrogenation of three.”
3. Logical rules are generated. An Inductive Logic Programming (ILP) algorithm[17] is used to generate rules that relate the presence or absence of structural features to activity. These rules can be readily understood by a chemist. An inductive logic programming algorithm[17] constructs logical rules that relate molecular substructure to activity. For each molecule in the training data set, every fragment is related to every other fragment of that molecule using the rule format “If Fragment A is *x* Ångströms from Fragment B, there will be some effect on activity” where *x* Ångströms is the distance measured between the two fragments in the 3D minimized structure. The rules are thus the leading and upper diagonal of an all-by-all fragment comparison matrix. The rules constructed for all the training data molecules are pooled and redundant rules (rules sharing the same two fragments where the difference in *x* between the two rules is less than one Ångström) eliminated.
4. Modules from the Java Chemistry Development Kit (CDK)[24] calculate a range of physicochemical descriptors for each molecule in the training data. These descriptors can be classified as being related to five aspects of chemistry:[25] size (molecular weight, mass distribution and atom counts), hydrophobicity (Log*P*), electronic (charge, polarisability, molecular orbital), hydrogen bonding (hydrogen donors and acceptors) and topological (calculated from graph representations of molecules).
5. The data is used to form a matrix for Support Vector Machine (SVM) analysis[26]: each molecule in the training set forms a vector (weighted by bioactivity), while the logical rules from step 3 and the physicochemical descriptors from step 4 are used as features. The SVM constructs a hyperplane classification relating rules to activity. The training data molecules are used as vectors, with the activity of the molecules as the vector weights and the rules (all the pairwise fragment-distance rules and the physicochemical descriptors) as vector features. The SVM package used was SVM-Light version 6.02[27,28] using the default linear kernel for all experiments described in this paper. The combined process of weighting ILP rules using SVM is known as SVILP.[16,29]
6. A dataset of screening data. The dataset used for screening in this paper was the “fragment-like” subset (*x*Log*P*≤3.5, Molecular weight≤250 Daltons, rotatable bonds≤5) given by the ZINC molecular database.[5,6] These criteria meet the definition of “fragment-like” molecules defined by Carr et al.[30]
7. The screening stage in which a quantitative prediction of activity is assigned to each molecule. To calculate a predicted activity value, the rules generated in step 3 that are fulfilled by the screened molecule are used as features for a vector to be multiplied by the matrix model generated by the SVM in step 5.

### 2.2 Using Chemical Reactions to Extend INDDEx

In order to extend INDDEx’s search into synthetic space, a module was added to perform virtual chemical reactions to generate predicted synthetic products, which could then be rescored using the INDDEx model. The method of virtual chemical reactions was designed to imitate the work process of a chemist using reactions to bring a molecule from hit to lead. Molecules with high potential for being active would be taken and derivatives made from them to see if it improves the predicted activity.

**Figure 2:**
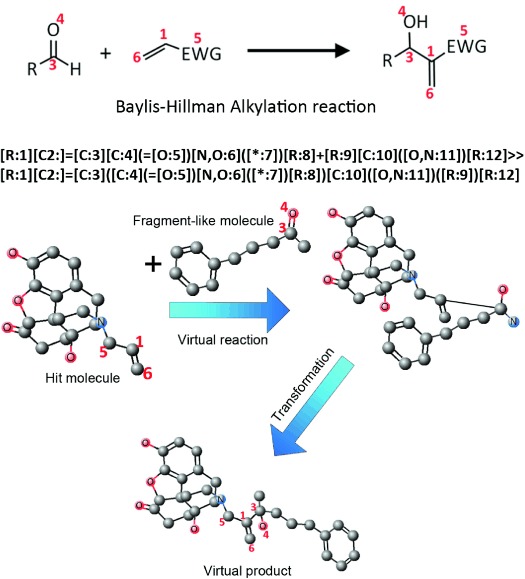
Top: The Baylis-Hillman Alkylation reaction, with the numbers that map to the atoms in the reactants and the corresponding atoms in the product. Middle: The Baylis–Hillman Alkylation reaction expressed as a SMIRKS. Bottom: An example of a reaction transformation process involving two example molecules undergoing the Baylis–Hillman Alkylation reaction and the subsequent transformation of the conformation.

The ChemAxon Reactor tool[8] was selected for the ease of integrating it into the existing INDDEx technology. ChemAxon’s Reactor contains a library of organic reactions, each with a SMIRKS (Simple Molecular Input Reaction Kinetic Strings) description[31] (an example of which is shown in Figure 2) which expresses reactions as transformations in mapped atoms and bonds between reactant and product SMILES (Simplified Molecular Input Line Entry System)[32,33] which defines the changes in atoms and bonds between the reactants and the product, and with a list of computationally formalised chemical rules that the reactants need to match in order for the reaction to take place. The ChemAxon rulesdefine physicochemical properties or take the form of SMARTS (SMiles ARbitrary Target Specification) expressions[31] that define areas of molecular substructure. Inclusive ChemAxon rules must be fulfilled in order for the reaction to be viable, and fulfilment of exclusive rules prevents reaction viability.

**Figure 3:**
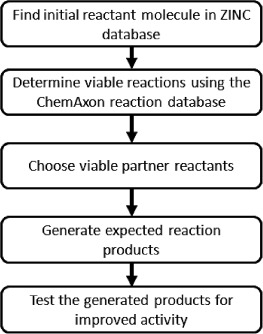
Flowchart summarising the steps in the INDDEx virtual reaction process.

ChemAxon reaction rules were integrated into INDDEx, allowing INDDEx to test potential reactants and produce virtual products. SMIRKS (Simple Molecular Input Reaction Kinetic Strings) were used to describe the structural transformations in the reactions.[31] Figure 3 summarises the steps involved in this process.

In the virtual reaction process, the first step is to take the molecule that is desired to be modified. This is termed the ‘initial reactant’. Viable reactions are found finding matching within the molecular structure to the molecular substructure in a SMIRKS reactant term. A search can then be made for viable ‘partner reactants’ that can take the place of the co-reactant in the reaction by looking for matches to the molecular substructure in the SMIRKS co-reactant term. The viability of the reaction can be found by applying the ChemAxon reaction rules to the two reactants. These reaction rules describe structural and physicochemical factors that are required for the reaction or would exclude the reaction from taking place. If the reaction is determined to be viable, the two reactants can be joined together according to the SMIRKS transformation to generate a list of bond and atom graphs of potential reaction products, which can be rescored using the INDDEx model. Secondary products of the reaction are discarded.

Due to the INDDEx rules specifying distance measures, the product must be formed into an approximately energetically favourable conformation with correct bond lengths and minimising of steric clashes. The when the reactants are joined together into a product, the 3D coordinates are transformed so that the newly formed bonds are the correct length and steric clashes between the reactant substructures are minimised. Figure 2 shows an example reaction and its description in SMIRKS format, along with an example of the whole reaction transformation process for the example reaction.

A concern of using addition reactions is that products of these reactions will become too large to act as effective drugs (95 % of drug weights fall below 625 Daltons).[34] In order to cut down on synthetically accessible space and search only a space of reasonably sized molecules, this assessment only searched through the ZINC fragment-like dataset for initial reactants and using the same dataset to search for partner reactants.

### 2.3 Weights of the ZINC Fragment-Like Molecules

The molecules from the fragment-like subset of the ZINC database used in these assessments contained 474 770 molecules. Their weights being 250 Daltons or less means that when two fragment-like molecules are joined by an addition reaction, the resultant product will never break the 500 Dalton weight criterion of the Lipinski “rule of five”[35] for the likelihood of a molecule being an orally bioavailable drug.

### 2.4 PLoRRS

INDDEx’s predicted scoring system is based on a matrix of logical rules (see the “INDDEx method” section), so changes in the molecular structure that lead to the fulfilment of more rules will lead to a higher predicted activity. Partial Logical-Rule Reactant Selection (PLoRRS) is a method developed to reduce the search space to explore by only considering molecules that are expected to yield a higher level of activity when modified with an organic reaction. When using the INDDEx model of activity, this corresponds to molecules where reactions could lead to the fulfilment of more INDDEx logical rules. PLoRRS calculates a measure of “fulfilment” from the logical rule-based model derived by INDDEx, based on how many of the rules for activity are fulfilled, to give an estimate of the potential activity of the product of a reaction, where molecules with a low fulfilment score are ones that have a higher potential for increased activity through having rules fulfilled. As well as reducing the search space of “initial reactants,” this also reduces the search space of corresponding “partner reactants,” as only partner reactants that match a cut-off number of the unfulfilled rules of the initial reactant are considered in virtual addition reactions (a cut-off of at least three rules was used throughout this study).

The PLoRRS method:

1. Finds the top 100 rules that have the highest positive correlation with the activity data and are in the format “Fragment A must be *x* Ångströms distant from Fragment B.”
2. For each initial reactant, a score is assigned based on how many of the rules from step one are half-filled (i.e. the molecule contains either Fragment A or Fragment B but not both).
3. Loops through the top 100 rules that are most positively correlated with activity.
4. If the rule is half-filled and the distance *x* between the fragments is greater than two Ångströms (to only consider rules that apply to two definitely separate fragments rather than one contiguous piece of substructure), one point is added to the PLoRRS score.
5. The molecules are then rank ordered from highest to lowest PLoRRS score (as the score is considered the molecule’s potential for increased activity with a reaction) and considered in turn until a cut-off for the number of molecules to be considered is reached.
6. Take each partner reactant in turn. The reactant fragments are checked against the half-filled rules from step 4 to see if they have a match for the unfilled half of the rule.
7. If the reactant has at least three rule fulfilments, then attempt a virtual reaction between the initial reactant and the partner reactant, and calculate a predicted activity using the INDDEx model if the reaction is successful.

## 3 Results

Two assessments were performed. The first assessment was to estimate the extent to which the virtual reactions opened up search space and the tractability of that space to brute-force search. The second assessment was to estimate the power of the PLoRRS method.

### 3.1 Assessment 1: Exploring the Extent to Which the Virtual Reactions Open up Search Space

To quantify an estimate of the size of search space accessible by using the virtual reactions, the following method was used:

1. One hundred molecules were randomly chosen from the unfiltered ZINC database, and another hundred from the ZINC database filtered for fragment-like molecules only. This generated a ZINC full-database sample and a ZINC fragment-like sample.
2. Each molecule in the two datasets was checked against the ChemAxon reactions to form a list of reactions it could participate in as a reactant.
3. For each reaction in the list, every molecule in the ZINC fragment database was checked for whether it could participate in the reaction.
4. Where both reactants can participate in a reaction, the reaction algorithm was run to generate and enumerate all products.

Table 1 shows the results of these tests.

**Table 1:** The number of average reactions, average reactant partners and average virtual products per molecule entered into the virtual reaction process, enumerated from 100 test molecules randomly selected from ZINC

	Number of molecules
Random test molecules used as initial reactants	100
Average reactions per molecule	2.28
Average reactant partners per molecule	27 228
Average total virtual products per molecule	53 450

The values in Table 1 allow an estimate to be made of the space opened up by utilising the virtual reactions. Multiplying the 53 450 average total virtual products per molecule with the number of molecules in the ZINC database (22 724 825 in the “all purchasable” set as of September 2014) gives an estimate of 1.21×10^12^. This compares with an estimate of 10^60^ for the whole of small-molecule space[1] (see Sect. 4, Discussion, for further comparisons).

### 3.2 Assessment 2: Estimating the Virtual Screening Power of the PLoRRS Method

#### 3.2.1 Quantifying the Power of the PLoRRS Method

**Figure 4:**
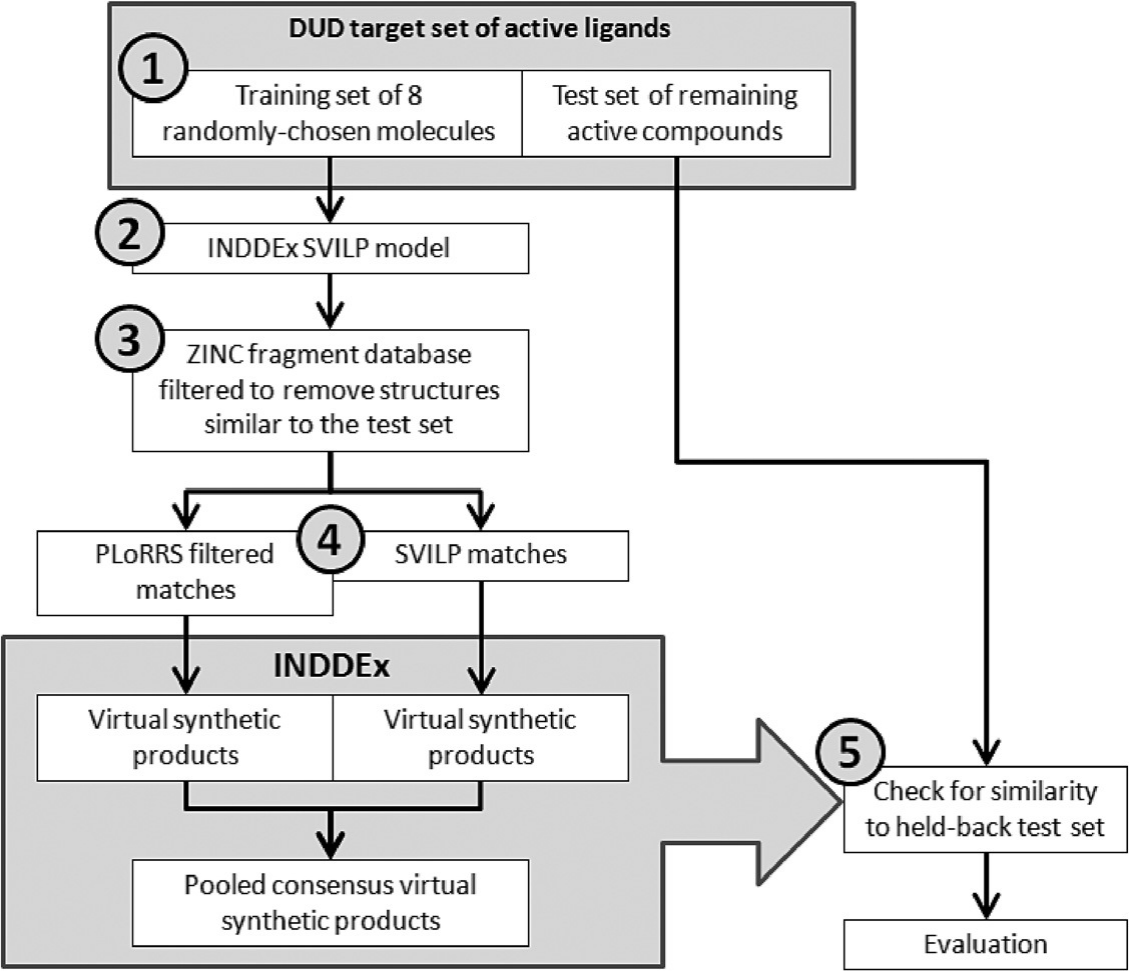
Flowchart showing the process used for the assessment of the power of the PLoRRS method against a naïve use of the SVILP method.

Figure 4 shows the method used to quantify the ability of using INDDEx with virtual reactions to search through virtual synthetic space and to compare the use of the SVILP model with the use of PLoRRS and using them both in consensus.

The null hypothesis was that PLoRRS does not enrich the virtual screening results when used independently or in consensus with the standard INDDEx SVILP model. The test dataset for the comparison were the 40 targets of the DUD database.[19] The list of DUD targets and their abbreviations is given in Appendix B of the supporting information. Each of the DUD targets comprises a set of known actives. The decoy sets in DUD were not used.

#### 3.2.2 Assessment Methodology

The procedure was:

1. For each of the forty targets, the known actives were divided into two sets: a training set of eight randomly selected active compounds and a held-back test set comprising the remaining actives. The eight active compounds were selected at random five times without replacement or until there were less than eight compounds remaining. This generated up to five datasets for each target.
2. INDDEx learned on a set of data and produced an SVILP model.
3. The ZINC fragment-like database was used as screening data, filtered for each test to exclude any molecules structurally similar to the molecules in the test set. A molecule was defined as structurally similar if it had an MCSS (Maximum Common Substructure) Tanimoto coefficient[36] of≥0.5 to any molecule in the test set. The assumption was that structurally similar molecules would have similar activity against the same target, though this is a generalisation and simplification of structure-activity relationships.
4. Three assessments were made: using the PLoRRS method, using only the SVILP model and using a consensus of the two. The PLoRRS method screens all the molecules in the database and then ranks them by PLORRS score, it then moves down the ranked list considering each one as an initial reactant and uses the list of unfulfilled PLoRRS rules to filter the list of partner reactants. The SVILP method screens all the molecules in the database and then ranks them by activity predicted by the SVILP model, it then moves down the ranked considering each one as an initial reactant and must consider every molecule in the database as a potential partner reactant. All products produced by these two methods were assigned a predicted activity by the SVILP model and ranked accordingly. The consensus method merges the list of products produced by the PLoRRS and SVILP methods to give a consensus result.
5. The virtual product molecules (ranked by activity) were compared for structural similarity against all molecules in the training and test sets, to test if INDDEx could use virtual reactions to generate molecules similar to the held-back actives.

#### 3.2.3 Exploration and Filtering of Synthetic Space

**Figure 5:**
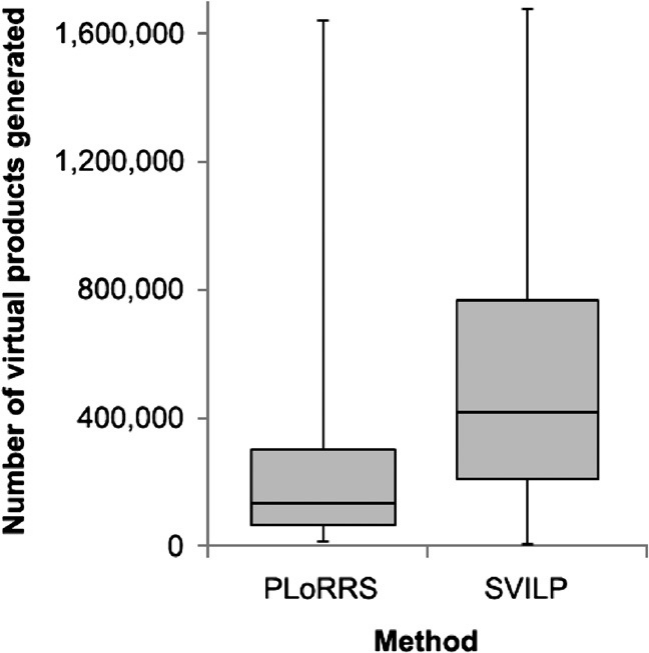
Box and whisker plots showing the distributions of the number of virtual products generated across the forty targets. The left-hand plot gives the distribution when using the PLoRRS method and the right-hand plot gives the distribution when using SVILP without PLoRRS. Box and whisker plots represent a five-number summary of a numerical set: the three horizontal lines making up the box mark the lower quartile, median and upper quartile of the set, and the whiskers extend to the maximum and minimum of the set.

Step 4 of the previous section generates a large number of virtual products per target. Figure 5 shows the distribution of the number of virtual products generated for each of the forty DUD targets. Because there is no filtering of partner reactants, the SVILP method generates far more virtual products, even though fewer initial reactants are considered.

In Assessment 2, the median reduction of partner reactant search space achieved by the PLoRRS filtration across the forty DUD targets was 97.4 %. Figure E1 in Appendix E of the Supporting Information shows the percentage amounts for the individual targets.

#### 3.2.4 Results of PLoRRS vs. SVILP vs. a Consensus of the Two

Figures C1 to C5 in Appendix C of the Supporting Information show retrieval graphs of molecules similar to held-back actives for each of the forty targets, and Table D1 in Appendix D of the Supporting Information tabulates the data from the retrieval graphs, giving the highest similarity to known actives achieved within the first 10, 100 and 1000 ranked molecules. As molecules with≥0.5 similarity were removed from the screening set, any similarity greater than 0.5 indicates the production of a molecule more similar to the held-back set than anything in the screening set. The number of targets that achieved similarities above 0.6, 0.7 and 0.8 are summarised in Table 2.

**Table 2:** Summary table of the results of the virtual screening power assessment

			Number of targets with a similarity value greater than		
Maximum similarity achieved by rank using:			0.6	0.7	0.8
	PLoRRS	10	1	0	0
		100	4	2	1
		1000	7	4	2
	SVILP	10	1	0	0
		100	3	1	0
		1000	3	1	0
	A consensus of PLoRRS and SVILP	10	2	0	0
		100	4	1	0
		1000	9	5	1

Table 3 applies McNemar’s test[37] to the data. These values result in a *p*-value of 0.0156 with a one-tailed test (using an exact binomial distribution) expecting the PLoRRS method to add additional power, or of 0.0313 with a two-tailed test.

**Table 3:** McNemar’s test comparing the successes of Naïve SVILP against the consensus method incorporating PLoRRS, defining success as greater than 0.6 similarity within the top 1000

	SVILP with PLoRRS success	SVILP with PLoRRS fail
Naïve SVILP success	3	0
Naïve SVILP fail	6	31

Table D2 in Appendix D conducts a more detailed statistical comparison, giving the one-tailed *p*-values when comparing the performances of the methods using the Mann–Whitney *U* statistical test.[38] These results indicate that using the consensus method is preferential to using either method individually, as using the consensus results in either an increased number of retrievals or the same amount.

#### 3.2.5 Case Studies of the Virtual Product Results

**Figure 6:**
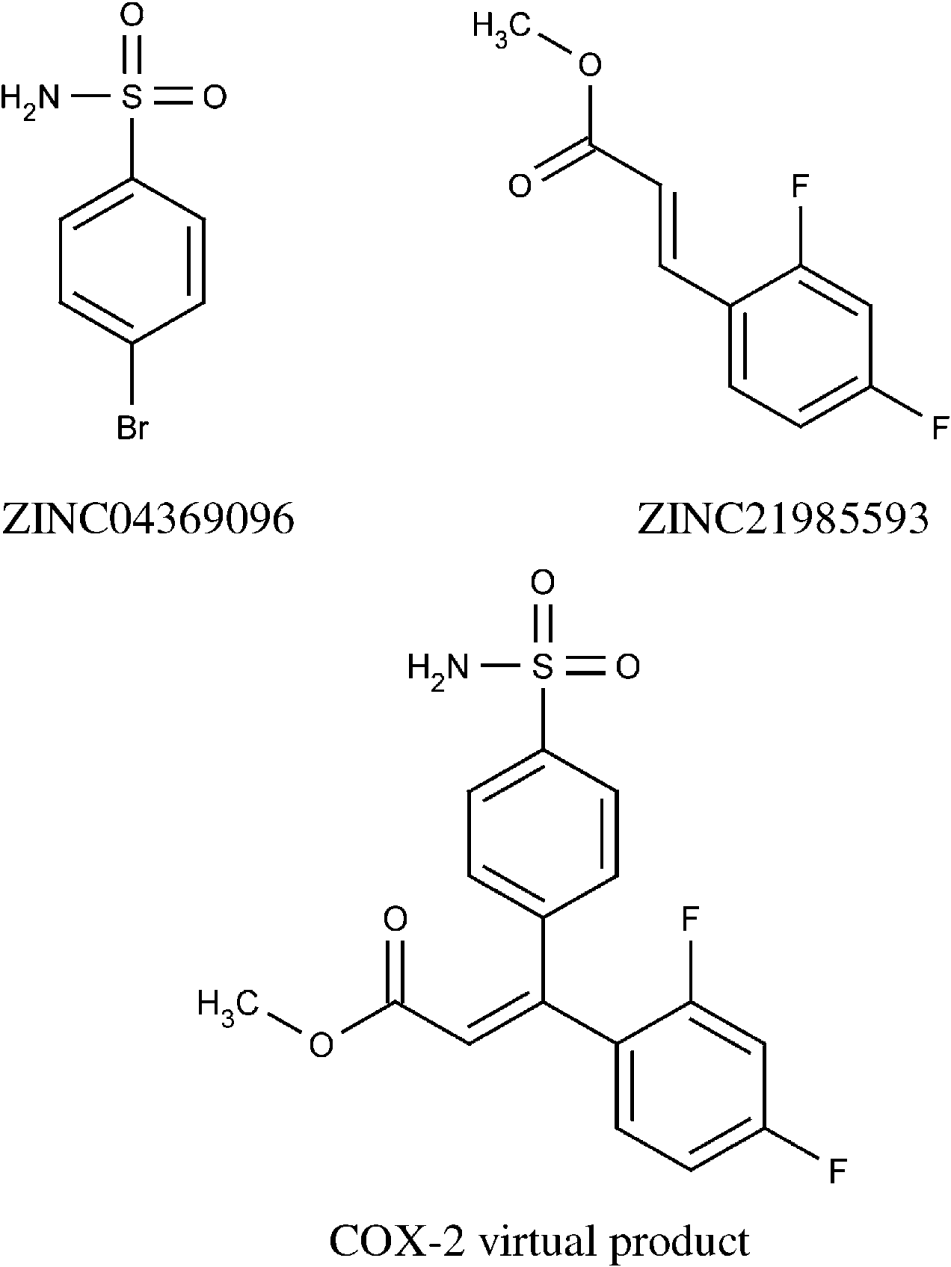
The initial reactant (top-left) and partner reactant (top-right) identified by the screening and the virtual product formed from them after undergoing the Heck reaction (bottom).

This section details two cases from the screening results of the COX-2 and EGFr targets where virtual products were formed that were highly similar to members of the held-back active sets. The COX-2 target screening formed a virtual product ranked 90^th^ in activity using the Heck reaction. The two fragment reactants and the virtual product formed are shown in Figure 6.

**Figure 7:**
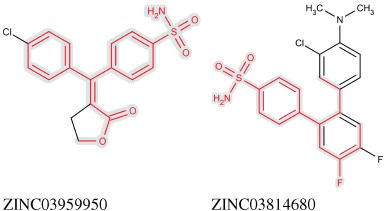
The closest match in the held-back actives, ZINC03959950 (left), and the closest match in the training data, ZINC03814680 (right), with the common substructure to the virtual product from Figure 6 highlighted in red on grey.

Figure 7 shows the most similar molecules to the virtual product from the held-back actives and the training data. Calculating the Tanimoto coefficient[36] from the common atom and bond substructure, it can be seen that the virtual product is much more similar to the most similar molecule in the held-back actives (Tanimoto of 0.834) than the most similar molecule in the training data (Tanimoto of 0.552). An additional similarity between the virtual product and the closest held-back active is that the fluorine substituted for chlorine are both halogens and the presence of fluorine in the closest training active provides additional evidence that this substitution would not hinder activity. The overall shape of all three molecules is similar, but the virtual product and the held-back active both have a 1,1-diphenylethene structure, while the training set active has a rigid *ortho*-terphenyl structure. This exhibits the way that ILP rules recognise the relational positioning of shape and feature characteristics in a way that simpler similarity measures do not.

**Figure 8:**
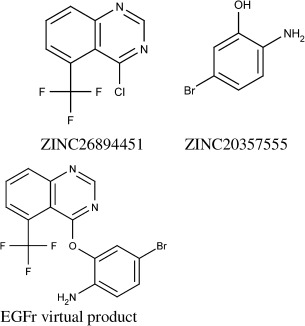
The initial reactant (top-left) and partner reactant (top-right) identified by the screening and the virtual product formed from them after undergoing the Ullmann condensation reaction (bottom).

The EGFr target screening formed a virtual product ranked 308^th^ in activity using the Ullmann condensation reaction. The two fragment reactants and the virtual product formed are shown in Figure 8.

**Figure 9:**
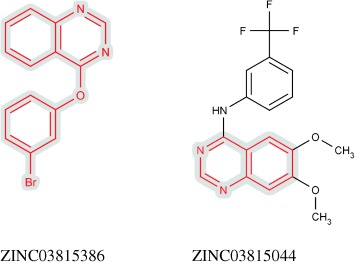
The closest match in the held-back actives, ZINC03815386 (left), and the closest match in the training data, ZINC03815044 (right), with the common substructure to the virtual product from Figure 8 highlighted in red on grey.

Figure 9 shows the most similar molecules to the virtual product from the held-back actives and the training data. As previously, the virtual product is much more similar to the most similar held-back active (Tanimoto of 0.791) than the most similar molecule in the training data (Tanimoto of 0.266). The structure of the held-back is a subgraph of the virtual product, with the virtual product having additional amine and trifluoromethyl groups. The trifluoromethyl occurs in the training active but not attached to the quinazoline, again exhibiting the ILP recognition of individual features.

#### 3.2.6 Case Studies of Blockbuster Drug Retrieval

Several of the molecules in DUD fit the definition of a “blockbuster” drug: a drug that generates over a billion dollars of revenue in a year. By examining the results, cases can be found in which blockbuster drugs were retrieved by INDDEx.

**Figure 10:**
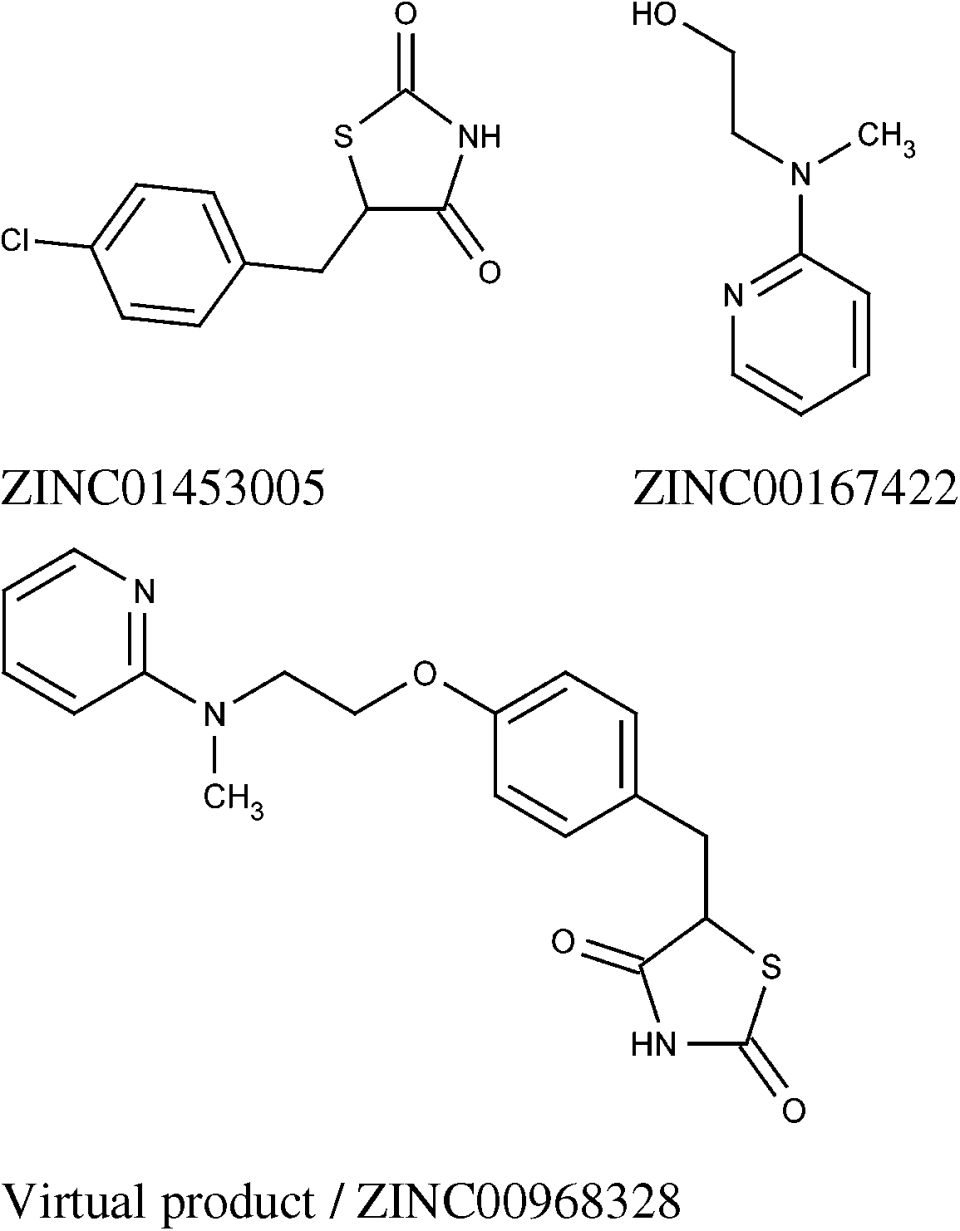
The initial reactant (top-left), partner reactant (top-right), and the resultant virtual product (bottom) formed with the Ullmann condensation reaction, and which is the chemical structure of Rosiglitazone.

The PPAR γ target dataset contains Rosiglitazone (ZINC00968328); a thiazolidinedione that binds to PPAR receptors, and sensitises them to insulin.[39] It was sold under the trade name Avandia by GlaxoSmithKline though sales fell after a meta-study linked it to an increased risk of heart attack.[40] The virtual reactions with the Ullmann condensation reaction generated the molecular structure of Rosiglitazone as shown in Figure 10.

#### 3.2.7 Filtering by Drug Likelihood

**Figure 11:**
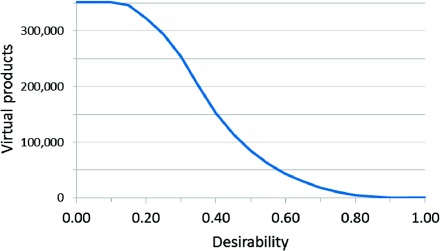
Line graph showing the number of virtual products as a higher desirability cut-off is used for filtration. Figures averaged over three representative targets (EGFr, COX-2 and P38).

A preliminary study was conducted into filtering by drug-likelihood. The most well-known measure of drug-likelihood is the “rule of five”.[35] On average, the virtual product molecules 62 % have no violations of Lipinski’s rule of five and 35 % have a single violation, which is allowed by the rule so it has little discrimination power here. More recently, a desirability score[34] has been developed to quantify the drug-likeness of a molecule based on Molecular Weight, Log*P*, H-bond Acceptors, and H-bond Donors. Desirability can be calculated for each virtual product and used to filter out molecules. Figure 11 shows the decrease in virtual products as a higher desirability cut-off is used. Bickerton[34] found that the mean desirability of approved drugs was 0.492. Setting the cut-off at 0.5 desirability removes 76 % of the virtual products, and a cut-off of 0.7 removes 95 %.

**Figure 12:**
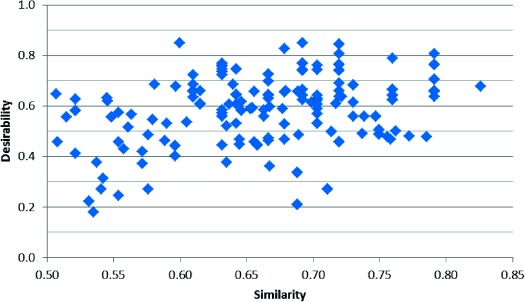
Desirability plotted against similarity for EGFr for the two hundred most active virtual product molecules in the consensus screening.

Figure 12 shows that looking at the top two hundred results of the consensus EGFr screening, 69 % are above 0.5 desirability and 21 % are above 0.7 desirability. Raising the desirability cut-off to 0.5 decreases the rank of the first similar hit (with 0.79 similarity) from 532^nd^ to 166^th^, and raising it to 0.7 decreases it to 12^th^.

Further work needs to be done to determine the best trade-off between desirability cut-off and retaining the molecules most likely to be active.

### 3.3 Speed and Timing Testing

All benchmarking was performed on a single core of an Intel i7-3820 CPU@3.60GHz, with all data reading/writing on a Samsung PM83 Solid state drive. The benchmark dataset was a pooled dataset of all actives and decoys in the DUD database.

The average time for virtual screening prediction was 3.6 ms per molecule, with descriptors being pre-calculated for all molecules. When using the virtual reactions, the time to produce the 3D structure of a single product molecule and calculate a predicted score was 107 ms. Exploring the products of a single molecule without PLoRRS filtering took 5719 seconds (95 minutes), and applying the PLoRRS filter reduces this to 148 seconds. To perform an energetic minimisation on the structure took an additional average 460 ms, so is generally not performed during a screening.

## 4 Discussion and Conclusion

This paper reports a software method of exploring lead space by performing virtual reactions of molecules predicted to lead to high activity. Existing methods of activity prediction (INDDEx) and reaction prediction (ChemAxon Reactor) were combined to produce a program that explores virtual synthetic space, and the PLoRRS algorithm has been developed to guide more effectively the choice of reactants and virtual reactions.

In Assessment 1, evaluations of a randomly selected sample of molecules were used to estimate the synthetically accessible search space opened up by the use of virtual reactions with INDDEx. The estimate was 1.21×10^12^ molecules, a space five orders of magnitude larger than the ZINC database, and the speed and timing estimates indicate that this space is so large as to be relatively intractable to a brute-force search, making clear the need for a method that can select reactants, implemented here as the PLoRRS algorithm. This potential search space was compared with previous estimates of chemical space in the literature. The estimate most often quoted for the space of all small molecules is 10^60^ after the calculation performed by Bohacek et al.[1] One subset of this space is the number of small molecules that it would be feasible to synthesise through organic chemistry, estimates for which vary between 10^20^[41] and 10^29^.[42] A second subset of this space is the number of drug-like molecules; estimates for which vary between 10^8^[43] and 10^30^.[2] These estimates compare with the sizes of existing databases of drugs as of August 2014: ChEMBL[44] contains 1.4 million compounds and ZINC[5] contains 16 million. There are currently 1584 FDA approved small-molecule drugs.[45]

The fragment-like database limits the products to a maximum size of 500 Daltons. While this is below the “rule of five” weight criterion,[35] it also limits the explorable space, potentially missing out larger molecules with high potential. A larger area of synthetic space could be searched by relaxing some of the criteria for the database used for the initial and partner reactants.

In Assessment 2, the virtual screening power of the virtual reactions system and the PLoRRS algorithm. As well as demonstrating the viability of incorporating the virtual reactions into a large-scale virtual screening, the results show a statistically significant advantage in using the PLoRRS algorithm in consensus with the INDDEx SVILP model over purely using the SVILP model with no means of directing reactant selection. The results were only significant when comparing the top 1000 ranked molecules. In actual practice, synthesising a thousand products would be prohibitive, so the section on filtering by drug likelihood describes a preliminary study to filter out highly-ranked inactives.

The virtual reactions were used to generate in the order of hundreds of thousands of products, but within this limitation, the naïve process was only able to explore the top 20 ranked initial reactants, but the PLoRRS filtering allowed the exploration of the top 150.

Assessment 2 makes two main assumptions. Firstly, that structural similarity to active molecules is correlated with activity. This is a simplification of structure-activity relationships, and, although it includes false positives (compounds with similar structure that would be inactive), ignores the space of true negatives (compounds with unrelated structure that would be active). Secondly, that the held-back molecules are accessible by the virtual reactions and reactants in the fragment database. Where the results do not show any virtual products with high similarity to actives, it could be due to a case where success is impossible with the reactants and reactions used.

**Figure 13:**
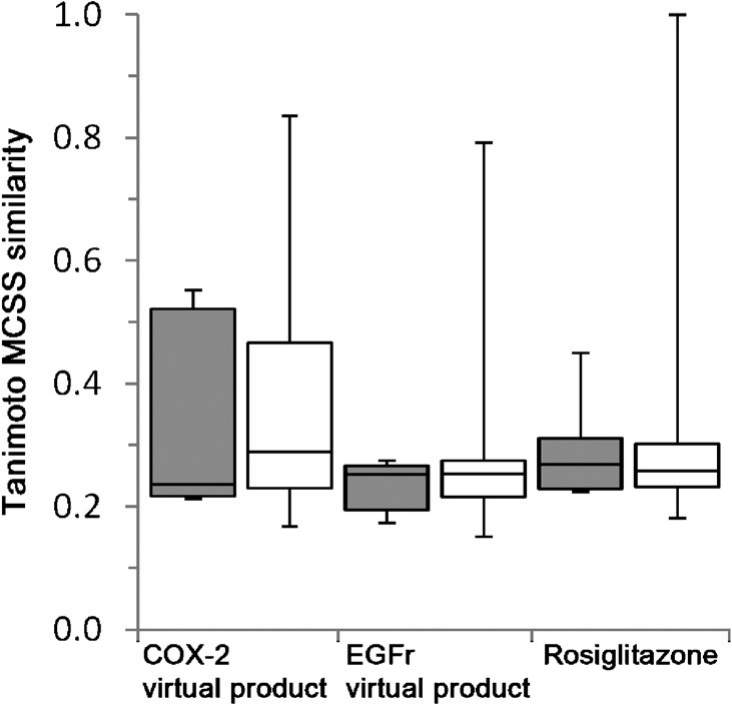
Box and whisker plots showing similarities of the three case study virtual products compared against all the molecules used in the training data (grey boxes) and the held-back data (white boxes).

The opening up of synthetic space provides additional value because the virtual molecules formed are likely to have novel structures, and the use of the ChemAxon rules means that each virtual product formed has been predicted to be synthetically accessible from two purchasable reactants and a standard organic reaction. Figure 13 compares the similarities of the three case study product molecules to the molecules used as training data, demonstrating how the virtual products formed are novel molecules distinct from the training data.

Assessment 2 provides a large underestimation of the method’s capabilities, because the only molecules considered active are the ones that were in the DUD active datasets and were held back. It cannot consider the possibility that the search may be identifying active molecules that are structurally distinct to the ones in the DUD datasets.

Further work would address the need for a system to filter molecules based on drug-likelihood profiles to arrive at a more manageable number of virtual products. The PLoRRS method used here operates on a simple count of the number of “top 100 ranked” rules half-fulfilled. A more nuanced version of PLoRRS would give additional weighting to the half-fulfilment of the higher-ranked rules. Further refinement might open the possibility of using two consecutive virtual reactions to open up an exponentially greater area of virtual space.

The virtual reaction module used in the work in this study only considers 39 of the most widely used organic reactions, but further reaction schemata can be added (ChemAxon Reactor has a database of 145 reactions).

## Supporting Information

As a service to our authors and readers, this journal provides supporting information supplied by the authors. Such materials are peer reviewed and may be re-organized for online delivery, but are not copy-edited or typeset. Technical support issues arising from supporting information (other than missing files) should be addressed to the authors.

miscellaneous_information
