# Supplementary material for: Incorporating Virtual Reactions into a Logic-based Ligand-based Virtual Screening Method to Discover New Leads
Source: Mol Inform. 2015 Mar 20;34(9):615–25. doi: 10.1002/minf.201400162 (PMC4641463; doi:10.1002/minf.201400162)
Supplement: Supplementary file 1 — miscellaneous_information [file minf0034-0615-sd1.pdf]

molecular informatics

models – molecules – systems

Supporting Information

© Copyright Wiley-VCH Verlag GmbH & Co. KGaA, 69451 Weinheim, 2014

Appendix A Virtual reactions encoded in INDDEx for this study

Table A1 lists the 26 reactions implemented as virtual reactions in INDDEx for the all the tests and assessments described in this paper. All were chosen as two-reactant addition reactions, and the rules describing elements of substructure and charge to include and exclude molecules as reactants in a viable reaction were taken from the ChemAxon Reactor database [8].

Table A1. List of the 26 virtual reactions provided by ChemAxon that were incorporated into INDDEx.

Acid azide synthesis	Houben Hoesch phenol acylation
Baylis-Hillman vinyl alkylation	Imino ester synthesis
Benary conjugated carbonyl synthesis	Isocyanate with nucleophile
Borch reductive amination	Knoevenagel condensation
Chan Lam coupling	Kumada coupling
Darzens epoxide synthesis	Perkin reaction
Fischer indole synthesis	Quellet chloroalkylation
Friedel-Crafts acylation	Ritter reaction of alcohols
Goldberg coupling	Ritter reaction of alkenes
Grignard addition to carbonyl compounds	Stille carbonyl synthesis
Guaresky Thorpe pyridone synthesis	Stille coupling
Heck reaction	Suzuki coupling
Henry nitro condensation	Ullmann condensation

Appendix B The DUD target sets

The DUD database contains forty target datasets, each consisting of ligand activity against a different protein target. Table B1 gives a list of all the protein targets, along with a description of each protein's role.

Table B1. A list of the 40 DUD targets, and the abbreviations used to refer to them.

Abbreviation	Full name	Abbreviation	Full name
ACE	Angiotensin-converting enzyme	HIVRT	HIV reverse transcriptase
ACHE	Acetylcholine esterase	HMGR	Hydroxymethylglutaryl-coenzyme-A reductase
ADA	Adenosine deaminase	HSP90	Human heat shock protein 90 kinase
ALR2	Aldose reductase	InhA	Enoyl-acyl carrier protein reductase
AmpC	AmpC-type beta-lactamase	MR	Mineralocorticoid receptor
AR	Androgen receptor	NA	Neuraminidase
CDK2	Cyclin dependent kinase 2	P38	P38 mitogen activated protein kinase
COMT	Catechol O-methyltransferase	PARP	Poly(ADP-ribose) polymerase
COX-1	Cyclooxygenase 1	PDE5	Phosphodiesterase V
COX-2	Cyclooxygenase 2	PDGFrb	Platelet-derived growth factor receptor kinase beta
DHFR	Dihydrofolate reductase	PNP	Purine nucleoside phosphorylase
EGFr	Epidermal growth factor receptor kinase	PPAR γ	Peroxisome proliferator-activated receptor gamma
ER agon.	Estrogen receptor agonist	PR	Progesterone receptor
ER antag.	Estrogen receptor antagonist	RXR α	Retinoic X receptor alpha
FGFr1	Fibroblast growth factor receptor 1	SAHH	S-adenosyl-homocysteine hydrolase
FXa	Factor Xa	SRC	Tyrosine kinase SRC
GART	Glycinamide ribonucleotide transformylase	thrombin	Thrombin
GPB	Glycogen phosphorylase beta	TK	Thymidine kinase (type-1)
GR	Glucocorticoid receptor	trypsin	Trypsin
HIVPR	HIV protease	VEGFr2	Vascular endothelial growth factor receptor kinase

Appendix C Individual retrieval graphs for each target

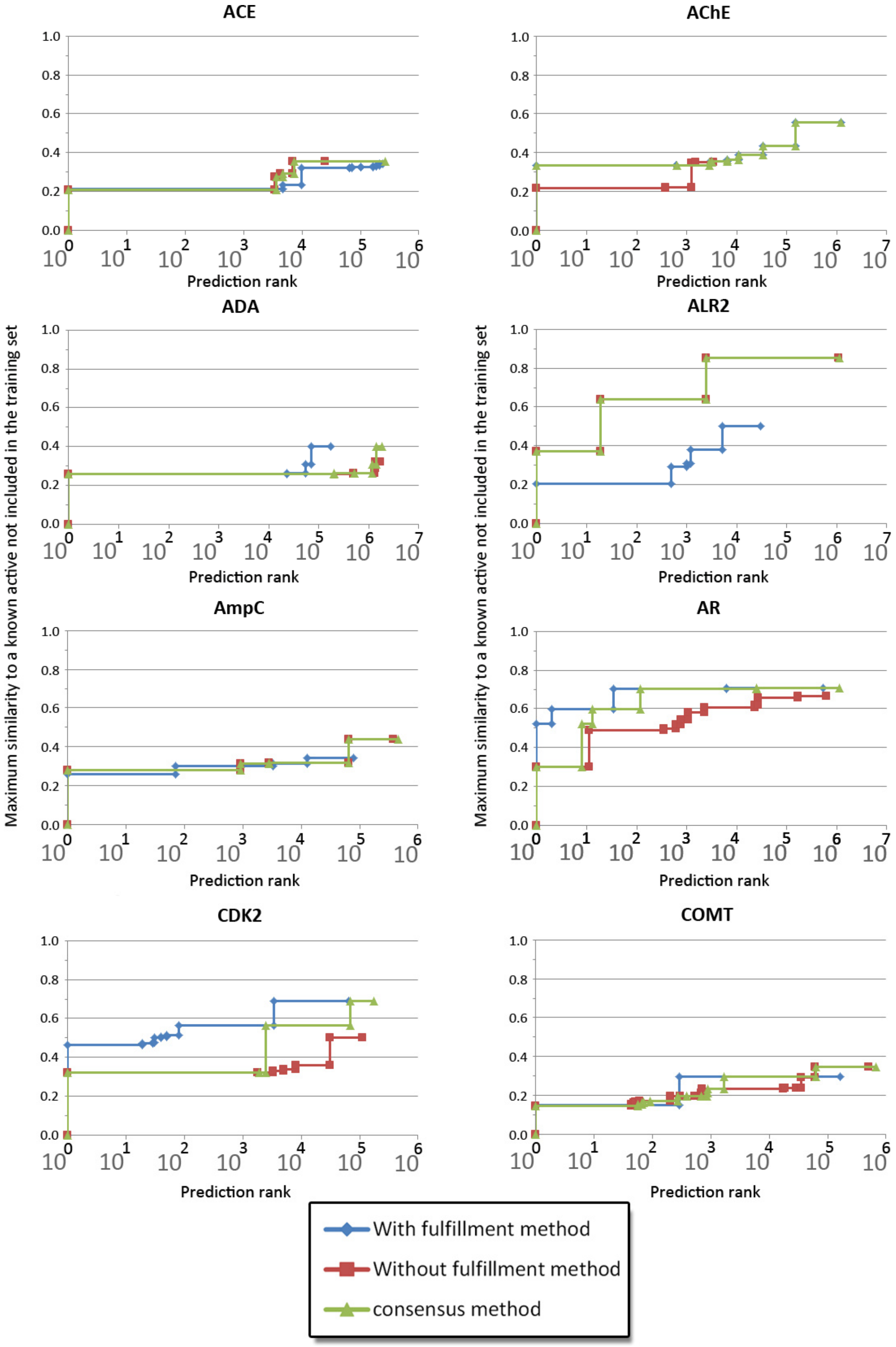

Figure C1. Graphs of retrieval at different similarity levels from ACE to COMT. For each graph, the *x*-axis is the rank order of all the generated virtual products, as predicted by the SVILP model. The *y*-axis is the maximum MCSS Tanimoto similarity of any virtual product, up to and including this rank, to any of the held-back actives.

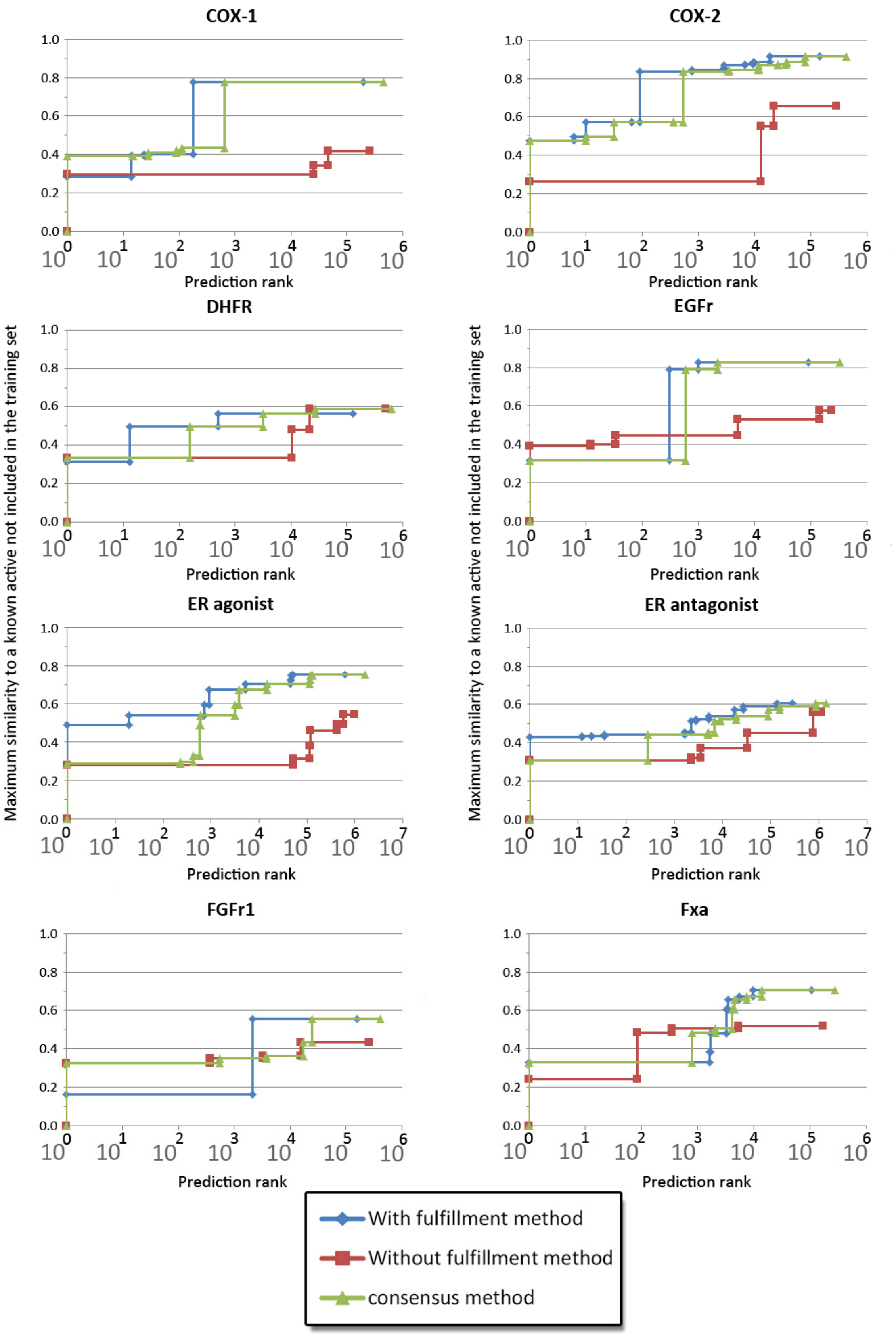

Figure C2. Graphs of retrieval at different similarity levels from COX-1 to Fxa. For each graph, the *x*-axis is the rank order of all the generated virtual products, as predicted by the SVILP model. The *y*-axis is the maximum MCSS Tanimoto similarity of any virtual product, up to and including this rank, to any of the held-back actives.

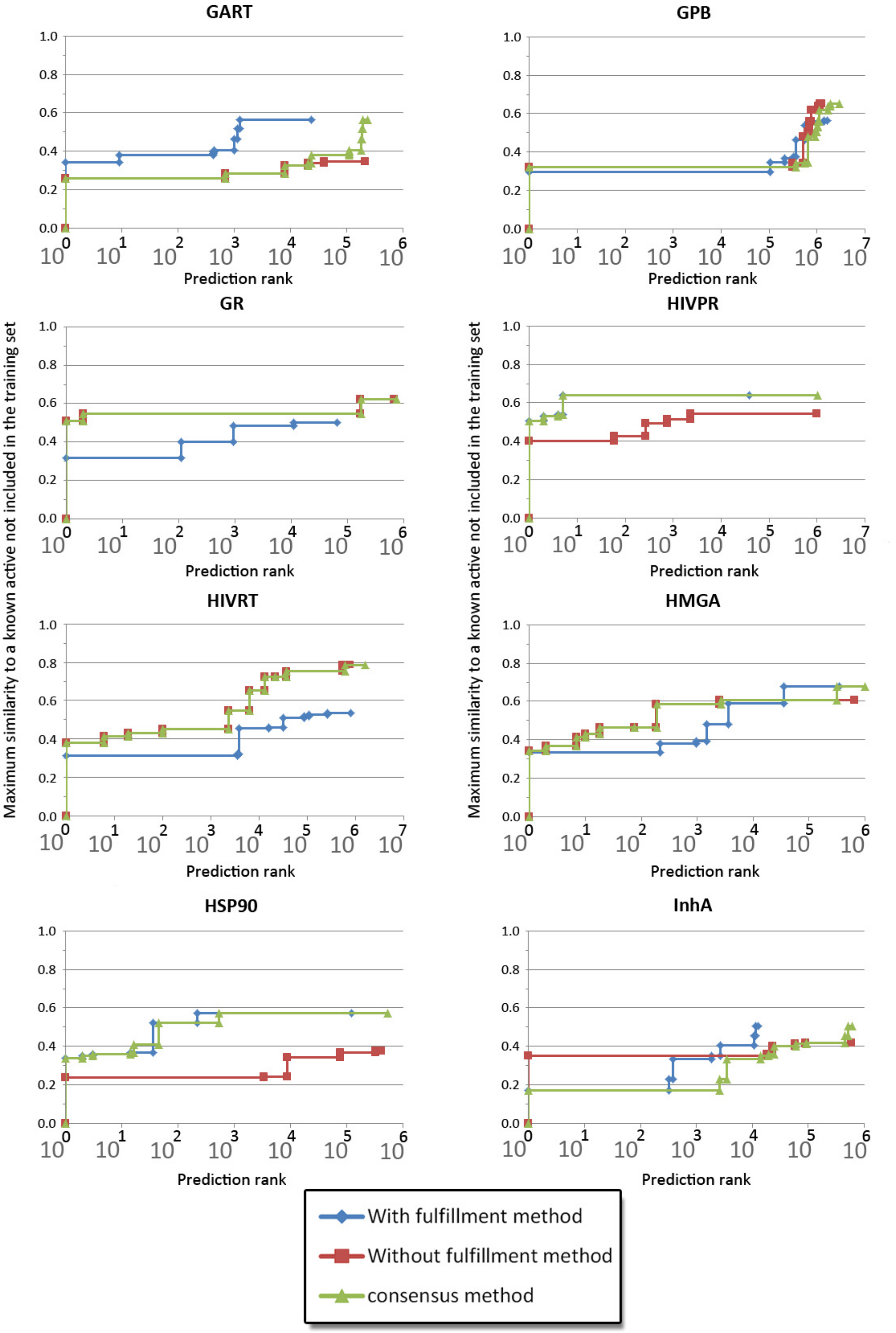

Figure C3. Graphs of retrieval at different similarity levels from GART to InhA. For each graph, the *x*-axis is the rank order of all the generated virtual products, as predicted by the SVILP model. The *y*-axis is the maximum MCSS Tanimoto similarity of any virtual product, up to and including this rank, to any of the held-back actives.

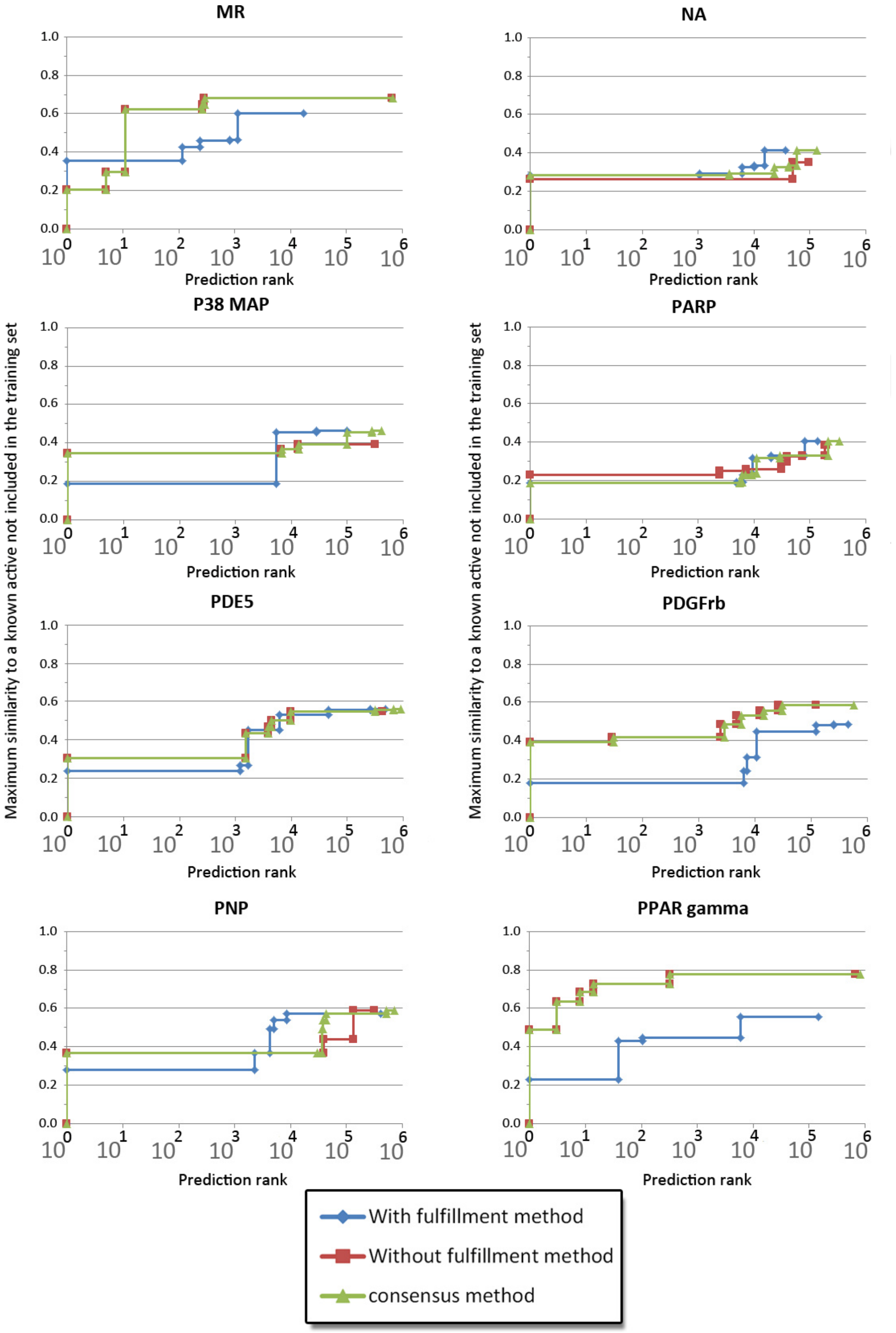

Figure C4. Graphs of retrieval at different similarity levels from MR to PPAR gamma. For each graph, the *x*-axis is the rank order of all the generated virtual products, as predicted by the SVILP model. The *y*-axis is the maximum MCSS Tanimoto similarity of any virtual product, up to and including this rank, to any of the held-back actives.

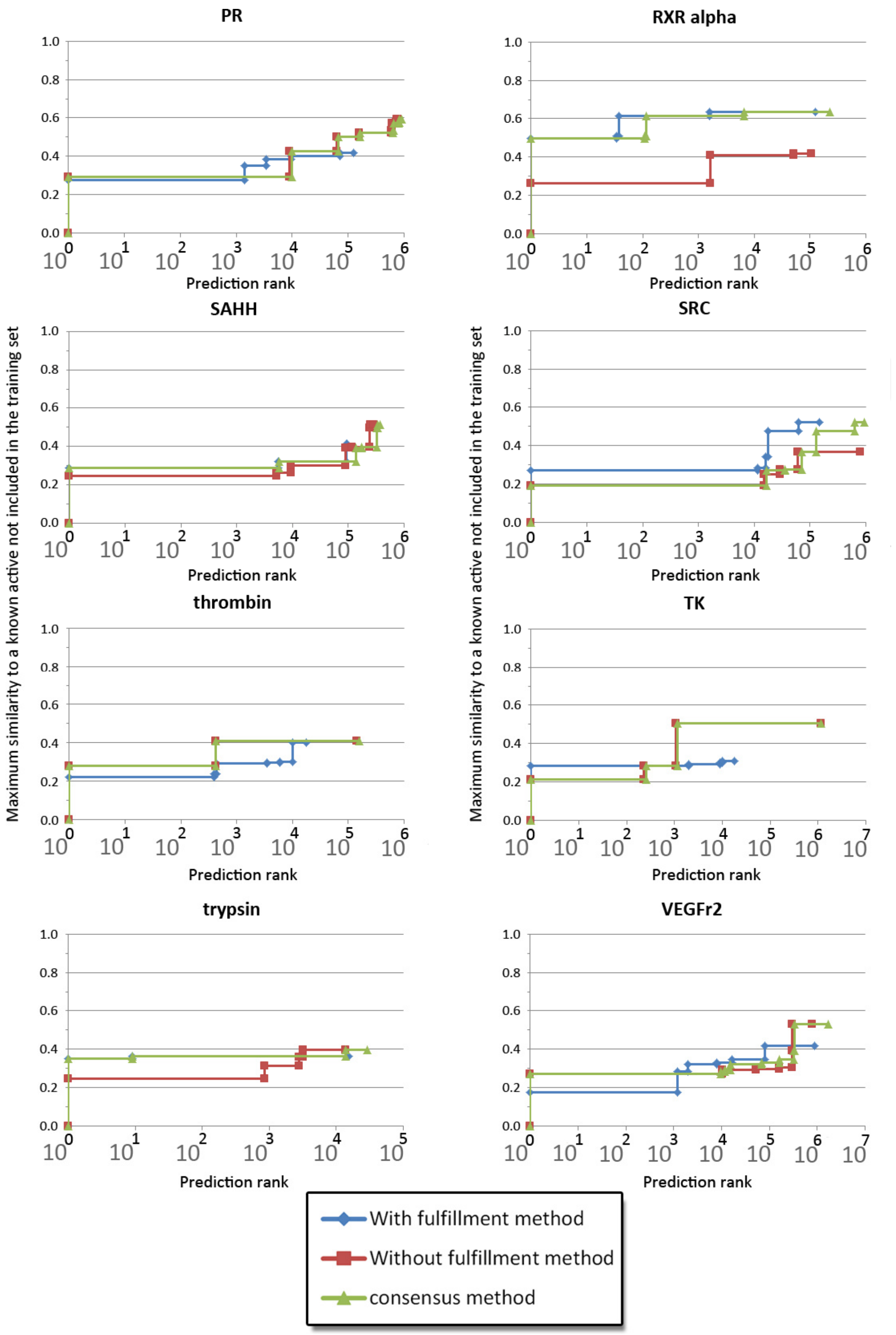

Figure C5. Graphs of retrieval at different similarity levels from PR to VEGFr2. For each graph, the *x*-axis is the rank order of all the generated virtual products, as predicted by the SVILP model. The *y*-axis is the maximum MCSS Tanimoto similarity of any virtual product, up to and including this rank, to any of the held-back actives.

Appendix D Results of the virtual screening power assessment

Table D1. Table of results of the virtual screening power assessment. The targets in the second column are from the DUD, and a description of each target's abbreviations and biological functions are defined in Appendix B. Mean similarity is the geometric mean of Maximum Common Substructure Tanimoto coefficients. Values are given to two decimal places, and similarities above 0.6 are highlighted in green, above 0.7 in orange, and above 0.8 in red.

ID	Target dataset	Active ligands	Mean similarity	Max similarity achieved by rank using PLoRRS			Max similarity achieved by rank using SVILP			Max similarity achieved by rank using consensus		
				10	100	1000	10	100	1000	10	100	1000
1	HIVRT	40	0.22	0.31	0.31	0.31	0.41	0.45	0.45	0.41	0.45	0.45
2	VEGFr2	74	0.20	0.18	0.18	0.18	0.27	0.27	0.27	0.27	0.27	0.27
3	CDK2	50	0.20	0.47	0.57	0.57	0.32	0.32	0.32	0.32	0.32	0.32
4	PDE5	51	0.20	0.24	0.24	0.24	0.31	0.31	0.31	0.31	0.31	0.31
5	COX-1	25	0.28	0.29	0.40	0.78	0.30	0.30	0.30	0.39	0.42	0.78
6	ALR2	26	0.26	0.21	0.21	0.29	0.37	0.64	0.64	0.37	0.64	0.64
7	PDGFrb	157	0.26	0.18	0.18	0.18	0.39	0.42	0.42	0.39	0.42	0.42
8	InhA	85	0.25	0.17	0.17	0.34	0.35	0.35	0.35	0.17	0.17	0.17
9	SRC	155	0.29	0.27	0.27	0.27	0.19	0.19	0.19	0.19	0.19	0.19
10	COMT	11	0.27	0.15	0.15	0.30	0.15	0.17	0.24	0.15	0.17	0.24
11	thrombin	65	0.35	0.22	0.22	0.30	0.28	0.28	0.41	0.28	0.28	0.41
12	ER agon.	67	0.35	0.49	0.54	0.67	0.28	0.28	0.28	0.29	0.29	0.54
13	AChE	105	0.35	0.33	0.33	0.33	0.22	0.22	0.22	0.33	0.33	0.33
14	trypsin	44	0.38	0.36	0.36	0.36	0.25	0.25	0.32	0.36	0.36	0.36
15	HIVPR	53	0.25	0.64	0.64	0.64	0.40	0.43	0.51	0.64	0.64	0.64
16	COX-2	348	0.28	0.57	0.83	0.85	0.26	0.26	0.26	0.50	0.57	0.83
17	FGFr1	118	0.33	0.16	0.16	0.16	0.33	0.33	0.35	0.33	0.33	0.35
18	ER antag.	39	0.30	0.43	0.44	0.44	0.31	0.31	0.31	0.31	0.31	0.44
19	ADA	23	0.42	0.26	0.26	0.26	0.26	0.26	0.26	0.26	0.26	0.26
20	GR	78	0.35	0.32	0.32	0.49	0.55	0.55	0.55	0.55	0.55	0.55
21	AmpC	21	0.41	0.26	0.30	0.30	0.28	0.28	0.32	0.28	0.28	0.32
22	HSP90	24	0.37	0.36	0.52	0.57	0.24	0.24	0.24	0.36	0.52	0.57
23	TK	22	0.55	0.29	0.29	0.29	0.21	0.21	0.28	0.21	0.21	0.28
24	P38 MAP	256	0.34	0.19	0.19	0.19	0.35	0.35	0.35	0.35	0.35	0.35
25	AR	74	0.31	0.60	0.70	0.70	0.30	0.49	0.54	0.53	0.60	0.70
26	MR	15	0.45	0.36	0.36	0.46	0.30	0.62	0.68	0.30	0.62	0.68
27	PR	27	0.34	0.28	0.28	0.28	0.29	0.29	0.29	0.29	0.29	0.29
28	PARP	33	0.50	0.19	0.19	0.19	0.23	0.23	0.23	0.19	0.19	0.19
29	EGFr	444	0.41	0.32	0.32	0.83	0.39	0.45	0.45	0.32	0.32	0.79
30	Fxa	142	0.27	0.33	0.33	0.33	0.24	0.49	0.51	0.33	0.33	0.49
31	PNP	25	0.50	0.28	0.28	0.28	0.37	0.37	0.37	0.37	0.37	0.37
32	ACE	49	0.35	0.21	0.21	0.21	0.21	0.21	0.21	0.21	0.21	0.21
33	HMGA	35	0.47	0.33	0.33	0.39	0.43	0.46	0.58	0.43	0.46	0.58
34	GPB	52	0.49	0.30	0.30	0.30	0.32	0.32	0.32	0.32	0.32	0.32
35	PPAR γ	81	0.44	0.23	0.43	0.45	0.68	0.73	0.78	0.68	0.73	0.78
36	NA	49	0.36	0.28	0.28	0.28	0.26	0.26	0.26	0.28	0.28	0.28
37	DHFR	201	0.35	0.31	0.50	0.57	0.33	0.33	0.33	0.33	0.33	0.50
38	GART	21	0.47	0.38	0.38	0.46	0.26	0.26	0.28	0.26	0.26	0.28

39	SAHH	33	0.44	0.29	0.29	0.29	0.25	0.25	0.25	0.29	0.29	0.29
40	RXR α	20	0.72	0.50	0.62	0.62	0.26	0.26	0.26	0.50	0.50	0.62
Number of targets with a similarity value greater than			0.6	1	4	7	1	3	3	2	4	9
			0.7	0	2	4	0	1	1	0	1	5
			0.8	0	1	2	0	0	0	0	0	1

Table D2. One-tailed p-values from the Mann–Whitney U test comparing the three methods when looking at the maximum similarity in both the top 100 and the top 1000 ranked virtual products. Values given to three decimal places.

	SVILP rank 100	Consensus rank 100	SVILP rank 1000	Consensus rank 1000
**PLoRRS rank 100**	0.464	0.214		
**SVILP rank 100**		0.203		
**PLoRRS rank 1000**			0.283	0.152
**SVILP rank 1000**				0.039

Appendix E Search space reduction

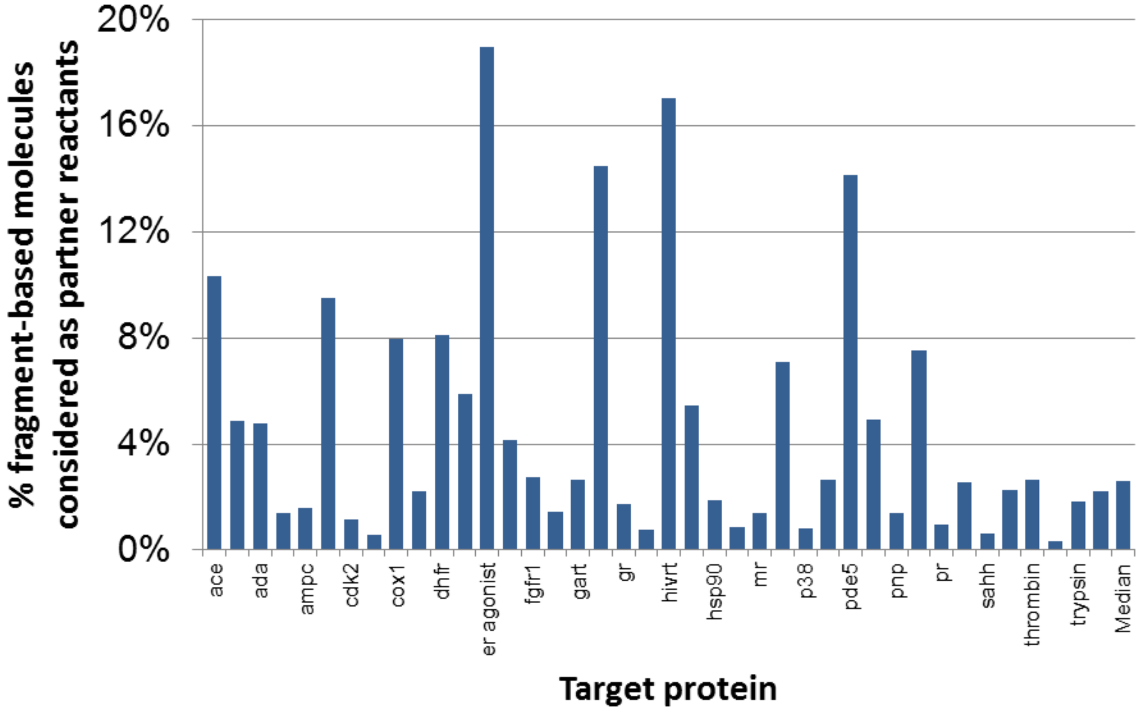

Figure E1. Bar chart showing, for each DUD target, the percentage of fragment-like molecules that are considered possible partner reactants after PLoRRS filtering. This chart shows what fraction of the database remains after applying the filter of at least three unfulfilled rules. The figures are calculated from the partner reactants considered for the first-ranked initial molecule screened for each target.
